# A preliminary epidemiological study of pinworm infection in Thaklong Municipal Early Childhood Development Center and Rangsit Babies’ Home, Pathum Thani, Thailand

**DOI:** 10.1186/s13104-018-3708-8

**Published:** 2018-08-20

**Authors:** Aree Taylor, Prasert Saichua, Pochong Rhongbutsri, Rattana Tiengtip, Sirima Kitvatanachai, Walter R. J. Taylor

**Affiliations:** 1 Department of Parasitology, Faculty of Medicine, Thammasat University, Khlong Neung, Pathum Thani, Thailand; 2 Laboratory Section, Faculty of Medicine, Thammasat University, Khlong Neung, Pathum Thani, Thailand; 3 Faculty of Medical Technology, Rangsit University, Khlong Neung, Pathum Thani, Thailand; 4 Mahidol Oxford Tropical Medicine Research Unit, Bangkok, Thailand

**Keywords:** Pinworm, Scotch tape technique, Socioeconomic status, Thailand

## Abstract

**Objectives:**

We investigated the prevalence and risk factors for *Enterobius vermicularis* in children at the Thaklong Municipal Early Childhood Development Center (TMECDC), and the Rangsit Babies’ Home (RBH) in Pathum Thani, Thailand using the Scotch tape method.

**Results:**

397 children aged 3–6 years were sampled (male = 198); 31 (7.8%) were *E. vermicularis* positive: 1 (TMECDC) and 30 (RBH). 264/397 (66.50%) of parents had incomes > 12,000 baht/month and 313/397 (78.84%) were educated from primary school to college. Univariate analysis identified (i) age 5–6 years, (ii) female sex, (iii) lower education of mother/father, (iv) being a house wife, (v) being a low income family, (vi) being resident in the orphanage, (vii) reporting anorexia and/or fever, and (viii) not washing their bottoms as factors for a positive slide. By logistic regression, education level and age group were independently associated with a positive Scotch tape result. Older children and higher family education had opposing associations with *E. vermicularis*. Strategies to control pinworm infection should focus on high-risk children in orphanages.

## Introduction

*Enterobius vermicularis* (pinworm), an intestinal nematode, remains a public health problem in many countries. It tends to be more common in school children in rural areas and in poorer urban areas [1–3]. Studies in school children have found different rates of pinworm infection. In Santiago city, Chile, infection rates were 20.9% [4], 0.62% in Taiwan [5], 58.88% in China [6] and, in one orphanage in Iraq, 84.31% [7].

*Enterobius vermicularis* has been reported in every part of Thailand, especially in children who live in rural areas [1, 2]. Epidemiological studies have reported variable rates of this parasite, e.g. in school children in Bang Khun Thian, the rate was 21.57% [8] and 38.82% of school children in Samutprakarn province [9]. The infection rate in kindergarten children in Chiang Mai Province was 45.38% [10] and 50.9% in kindergarten children in Khon Kaen Province [11]. No pinworm was found in a kindergarten that is used by educated lecturers at one Thai university, Thammasat, in the northern Bangkok conurbation [12]. One study in an orphanage in the same area found a rate of 15.95% [13]. Three studies from urban slum areas in Thailand report high rates of between 51 and 65%, in young children [11, 14, 15], rates higher than the 7% found in Karen hill tribes of NE Thailand [16].

Compared to other intestinal parasites, transmission of pinworm is limited because their eggs are unable to survive in the environment. The main routes of infection are autoinfection from eggs or larvae deposited on the anus [3], contamination from fomites like bed sheets, pyjamas, door handles, and inhalation of eggs from hands, bed mattresses or dust. As a result, infections tend to be limited to families and individuals in close proximity like nurseries and boarding schools.

Many cases of *E. vermicularis* infection are asymptomatic but when symptomatic, the most common symptoms include intense itching and inflammation of the anal/or vaginal areas. These symptoms may be accompanied by intestinal irritation, mild nausea or vomiting, irritability, and difficulty sleeping. Heavy infections may cause intestinal inflammation, secondary bacterial infection, abdominal pain and appendicitis. In one series of 405 appendicitis patients, 26 (6.3%) had pinworm [17]; the finding of pinworms in inflamed appendix walls supports the notion that pinworms can cause appendicitis [18, 19].

Other deleterious effects of pinworm include secondary bacterial perineal infections, invasion of organs such as vagina, uterus and gall bladder [20]. In small children, pinworm can reduce intestinal absorption of digested food and lead to malnutrition that in turn impedes growth and development.

Treating pinworm infection effectively requires treating all close contacts. Mebendazole is a drug of choice. Personal hygiene is also important for prevention and control such as washing hands before eating, keeping nails short, cleaning sleeping areas and drying mattresses under sunlight in order to destroy parasite eggs.

Several factors are associated with pinworm infection such as age, gender, and behaviour, e.g. biting or sucking fingers, and poor personal hygiene practices, notably scant attention to hand washing. Lower parental socioeconomic status and poorer education are well-described risk factors [8, 9, 21]. Parents may not have sufficient time to take care of their children and may not be aware of the importance of personal hygiene.

Data on the risk factors for pinworm infection in Thailand are limited. We, therefore, conducted a study to ascertain the prevalence and risk factors of pinworm infection and report our results herein. Such data will be useful for targeting control measures.

## Main text

### Methods

The study took place in December 2015 at two centres: the Thaklong Municipal Early Childhood Development Center (TMECDC), a well-baby clinic, and the Rangsit Babies’ Home (RBH), an orphanage, both located in the northern Bangkok conurbation of Pathum Thani province. The TMECDC is located some 2 km from the Thammasat University (TU) and serves the local paediatric population (age up to 7 years) of the Klong municipality. The RBH is an orphanage, situated some 8 km from TU; it houses approximately 300 children from 6 months to 7 years of age. Most are either orphans, former street children, caught up in custody battles, or abandoned.

These centres were selected because of their close proximity to TU and represented two different paediatric populations.

The parents of children attending the TMECDC were sent a consent form, questionnaire and a letter explaining the purpose and nature of the study, including that the results would be communicated only to them and that all collected data were confidential. For the children in the RBH, the authorities are their legal guardian so the same information and forms were sent to them and the head of the RBH signed the consent forms. If parents and authorities allowed the child to be recruited into this study, they were to return the signed consent form and the completed questionnaire.

We sampled children once in the morning at both centres using the Scotch-tape technique. Mothers and staff had been told not to wash the child’s bottom on the day of sampling. Slides were examined using standard light microscope and 20% of negative slides were re-examined for quality control. Symptoms (e.g. anal itching, malaise) and risk factors (e.g. household income, parental occupation) were collected using a structured questionnaire.

The Scotch-tape technique [22, 23] involves sticking Scotch-tape on to a tube and using this to touch the skin around the anus to pick up eggs. The contents of the tape are then transferred onto a glass slide and examined using standard light microscope. Oval pinworm eggs measure 50–60 × 20–30 μm and contain an embryo. When children tested positive, the parent or staff were informed of the result and were given an information sheet about pinworm and its prevention.

Because this was a preliminary study, we did not perform a sample size calculation. However, a sample size of 385 would allow us to detect a prevalence rate of 10% with a precision of 3%. The data from the structured questionnaires were checked for completeness and then double-entered and cleaned using Epidata v4 (http://www.epidata.dk/). Data were analysed with Stata v6 (Stata Corporation, TX, and USA). The significance of differences in categorical data was examined using Chi square tests or Fisher’s exact test, as appropriate. Logistic regression was used to assess the independence of explanatory variables for pinworm infection.

### Results

207 children from the TMECDC participated in the project. There are 101 males (48.79%) and 106 females (51.21%). Table 1 shows that the majority (94.69%) of children was in the age group of 3–4 years. Parents were educated mostly to secondary level (51.69%) and worked as company employees (51.21%) with monthly incomes exceeding 14,000 baht (48.3%).

**Table 1:** Characteristics of study subjects

Characteristics	TMECDC, n = 207 (%)	RBH, n = 190 (%)	P value
Age group (years)			
≤ 2	11 (5.3)	43 (22.63)	0.000
3–4	196 (94.69)	73 (38.42)	
5–6	0 (0)	62 (32.63)	
7–8	0 (0)	12 (6.32)	
Sex			
Female	106 (51.21)	93 (48.95)	0.653
Male	101 (48.79)	97 (51.05)	
Education			
Primary	8 (3.86)	0 (0)	0.000
Secondary	107 (51.69)	71 (37.37)	
College	39 (18.84)	119 (63.63)	
Bachelor	47 (22.71)	0 (0)	
> Bachelor	6 (2.9)	0 (0)	
Occupation			
Housewife	21 (10.14)	26 (13.68)	0.000
Government officer/State employee	19 (9.18)	164 (86.32)	
Trade/business	51 (24.64)	0	
Company employee	106 (51.21)	0	
Labourer/technician	9 (4.35)	0	
Other	1 (0.48)	0	
Income			
< 8000 baht	8 (3.86)	63 (33.16)	0.000
8001–10,000	33 (15.94)	0	
10,001–12,000	29 (14.01)	0	
12,001–14,000	37 (17.87)	103 (54.21)	
> 14,000	100 (48.31)	24 (12.63)	
History of stool examination			
Yes	3 (1.45)	0	0.096
No	204 (98.55)	190 (100)	
Bottom cleaning before sample collection			
Wash	170 (82.13)	36 (18.95)	0.000
Not wash	37 (17.87)	154 (81.05)	
Eggs found			
Yes	1 (0.50)	30 (15.80)	0.00
No	206 (95.50)	160 (84.20)	

Children from RBH numbered 97; sexes were approximately equal. The majority of children were also in the age group of 3–4 (38.42%) and 5–6 (32.63%) years. Parents were educated mostly to college level (63.63%), none had an undergraduate degree, most were government or state employees whose monthly income exceeded 12,000 baht (Table 1).

**Fig. 1:**
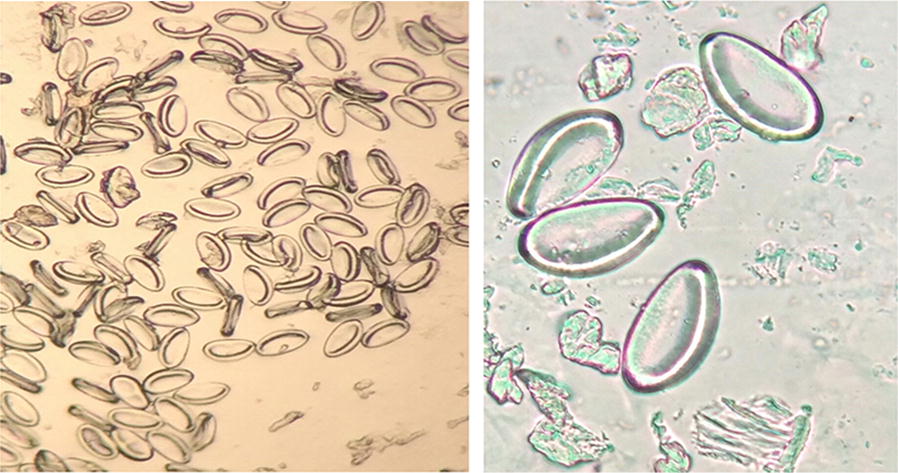
*Enterobius vermicularis* eggs found in this study

The results of microscopic examination of 40× magnification showed that the appearance of the eggs of the parasite was similar (D), as shown in Fig. 1.

Of a total 397 children, 31 children were infected (7.81%): one in the TMECDC and 30 in the RBH. Table 2 shows significant relationships (univariate analysis) between infected group and non-infected groups: age, sex, education level, occupation, income, and residing in the orphanage. In addition, anorexia, fever and washing the child’s bottom before the examination were also significant factors.

**Table 2:** Factors related to parasitic infection

	Infected group, n = 31 (%)	Non-infected group, n = 366 (%)	P value
Age group (years)			
≤ 2	0 (0)	54 (14.75)	0.000
3–4	6 (19.35)	263 (71.86)	
5–6	21 (67.74)	41 (11.2)	
7–8	4 (12.9)	8 (2.19)	
Sex			
Female	22 (70.97)	177 (48.36)	0.016
Male	9 (29.04)	189 (51.64)	
Education			
Primary	0 (0)	8 (2.19)	0.003
Secondary	23 (74.19)	155 (42.33)	
College	8 (25.81)	150 (41.0)	
Bachelor	0 (0)	47 (12.84)	
> Bachelor	0 (0)	6 (1.64)	
Occupation			
Housewife	10 (32.26)	37 (10.11)	0.000
Government officer/State employee	20 (64.52)	163 (44.54)	
Trade/business	1 (3.22)	50 (13.66)	
Company employee	0 (0)	106 (28.96)	
Labourer/technician	0 (0)	9 (2.46)	
Other	0 (0)	1 (0.27)	
Income			
< 8000 baht	23 (74.19)	48 (13.11)	0.000
8001–10,000	1 (3.23)	32 (8.74)	
10,001–12,000	0 (0.00)	29 (7.92)	
12,001–14,000	7 (22.58)	133 (36.34)	
> 14,000	0 (0.00)	124 (33.88)	
Stool examination			
Yes	0 (0)	3 (0.82)	0.613
No	31	363 (99.18)	
Crying at night			
Yes	0 (0)	35 (9.56)	0.071
No	31 (100)	331 (90.44)	
Bottom itching			
Yes	0 (0)	31 (8.47)	0.091
No	31 (100)	335 (91.53)	
Diarrhoea			
Yes	0 (0)	36 (9.84)	0.067
No	31 (100)	330 (90.16)	
Anorexia			
Yes	0 (0)	68 (18.58)	0.008
No	31 (100)	298 (81.42)	
Fever			
Yes	1 (3.23)	98 (26.78)	0.004
No	30 (96.77)	268 (73.22)	
Bottom cleaning			
Wash	0 (0)	206 (56.28)	0.000
Not wash	31 (100)	160 (43.72)	
Places			
TMECDC	1 (3.23)	206 (56.28)	0.000
RBH	30 (96.77)	160 (43.72)	

Logistic regression identified a higher educational level as protective against pinworm [odd ratio (OR) 0.189 (95% CI 0.07–0.51), P = 0.001] but a higher age group was associated with pinworm infection [OR 2.011 (1.001–4.04), P = 0.049].

### Discussion

This study has found that children from the orphanage, a socially disadvantaged group, accounted for the vast majority of pinworm infections. Pinworm infections in our study were associated with a lower family income and higher age.

*Enterobius vermicularis* infection is still reported in many countries because it occurs often in children who live together in close communities like orphanages and overcrowded households and is transmitted easily in such environments. Several studies from Thailand report high prevalence rates of infection in high density (> 50%) or slum (85%) areas of urban Bangkok [15], a kindergarten (> 50%) in Khon Kaen Province [11] but lower rates (7–15%) in the Karen hill tribes [16, 24].

This study was performed using the Scotch tape technique, a well know, widely used, easy, safe and reliable technique [7, 25]. In this study, 31 children were infected with pinworm, just under 8%. However, only one case from the total number of 207 children (0.50%) was found at the TMECDC and 30 cases (15%) from the RBH. This 15% rate is almost identical to the rates (16%) found at two other orphanages in Bangkok [26] and in rural based Karen children at Mae Chaem, Chiangmai Province [24] and is a little lower than one study (21%) in Chilean schoolchildren [4].

In our study, most infected children (about two-thirds) were older, aged 5–6 years, which is consistent with many other studies [13, 16, 19, 21, 27–32]. However, in one survey in Klong Toey, a crowded slum based community, 85% of infections were in children aged 8–9 years [15]. We also reconfirmed the importance of lower socioeconomic status as a risk factor for pinworm infection [8, 33, 34].

We sampled children in the morning at the TMECDC, consistent with other studies, and parents and staff were asked not to wash the child’s bottom on the day of sampling. However, most children washed their bottom before sampling and this may explain partly the low rate of detection at the TMECDC. By contrast, we sampled children at the RBH before the children showered, increasing our chances of obtaining a positive sample. In retrospect, it may have been better to have trained the parents to take the Scotch sample at home. Moreover, taking three samples instead of one would have increased our sensitivity, as found in one study in Malaysia [29], and sampling fingernails may also have increased our sensitivity.

The large difference in pinworm detection rates may also be due to greater overcrowding in the orphanage and increased chances of contact between infected and uninfected children. This calls for greater efforts to target control intervention in high-risk groups such as promoting good hygiene for children, cutting nails regularly, and health education for care staff, teachers and parents.

## Limitations

This small study was conducted in two types of institutions looking after children. We only sampled children once and so the sensitivity for detecting pinworm infection was reduced.

## Abbreviations

TMCDC: Thaklong Municipal Early Childhood Development Center
RBH: Rangsit Babies’ Home
